# Pyroptosis in asthma: inflammatory phenotypes, immune and non-immune cells, and novel treatment approaches

**DOI:** 10.3389/fphar.2024.1452845

**Published:** 2024-11-13

**Authors:** Yuqiu Hao, Wenrui Wang, Lin Zhang, Wei Li

**Affiliations:** ^1^ Department of Respiratory and Critical Care Medicine, The Second Hospital of Jilin University, Changchun, Jilin, China; ^2^ Department of Hepatopancreatobiliary Medicine, Digestive Diseases Center, The Second Hospital of Jilin University, Changchun, Jilin, China

**Keywords:** asthma, pyroptosis, cell, inflammation, therapy

## Abstract

Pyroptosis is a form of inflammatory programmed cell death, and is activated by pathogen infections or endogenous danger signals. The canonical pyroptosis process is characterized by the inflammasome (typically NLRP3)-mediated activation of caspase-1, which in turn cleaves and activates IL-1β and IL-18, as well as gasdermin D, which is a pore-forming executor protein, leading to cell membrane rupture, and the release of proinflammatory cytokines and damage-associated molecular pattern molecules. Pyroptosis is considered a part of the innate immune response. A certain level of pyroptosis can help eliminate pathogenic microorganisms, but excessive pyroptosis can lead to persistent inflammatory responses, and cause tissue damage. In recent years, pyroptosis has emerged as a crucial contributor to the development of chronic inflammatory respiratory diseases, such as asthma. The present study reviews the involvement of pyroptosis in the development of asthma, in terms of its role in different inflammatory phenotypes of the disease, and its influence on various immune and non-immune cells in the airway. In addition, the potential therapeutic value of targeting pyroptosis for the treatment of specific phenotypes of asthma is discussed.

## Introduction

Based on the type and number of inflammatory cells in induced sputum, asthma is categorized into four inflammatory phenotypes: eosinophilic, neutrophilic, mixed granulocytic, and paucigranulocytic asthma. Both eosinophilic and neutrophilic asthma can involve significant airway inflammation and tissue damage. Eosinophilic asthma is often sensitive to oral and inhaled glucocorticoids (Hussain et al., 2024), while severe neutrophilic asthma is resistant to glucocorticoid therapy (Zhang et al., 2022). Thus, the effectiveness of asthma medications depend on the specific inflammatory phenotype of the disease. Understanding the molecular mechanisms that contribute to the development of different asthma phenotypes is critical for identifying new therapeutic targets for effective asthma treatment.

**FIGURE 1:**
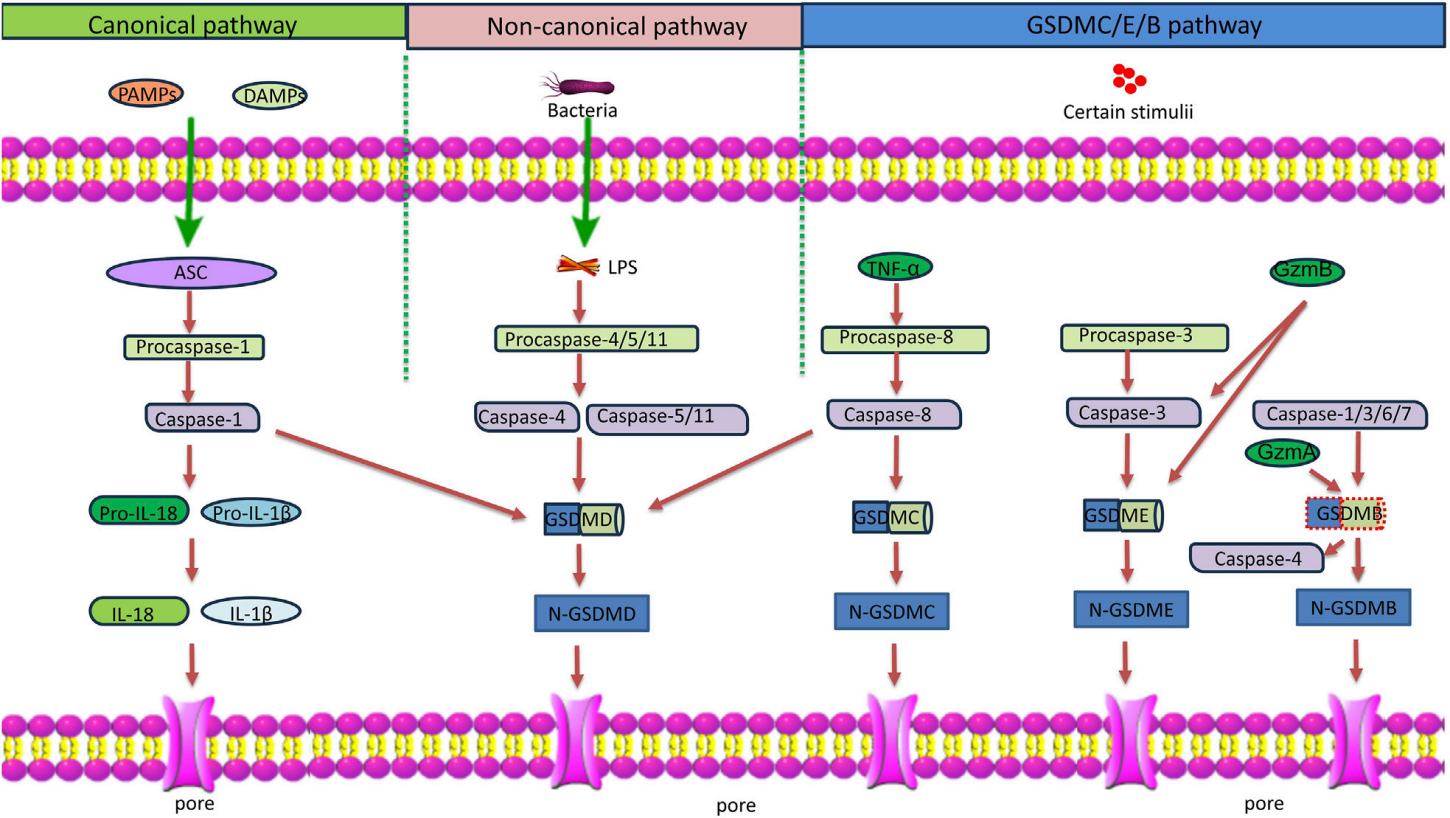
Schematic diagram of the pyroptosis pathways. Classical pyroptosis pathway: PAMPs and DAMPs activate inflammasomes and caspase-1. Caspase-1 cleaves GSDMD and pro-IL-1β/18. N-GSDMD perforates the cell membrane by forming nonselective pores, causing cell lysis and death. IL-1β/18 are released through the pores formed by N-GSDMD. Non-classical pyroptosis pathway: LPS derived from bacteria recognizes and activates caspase-11/4/5, leading to GSDMD cleavage, and triggering pyroptosis. In addition, caspase-4 also processes IL-18. GSDMC/E/B pathway: GSDMC: TNF-α induces the activation of caspase-8, which in turn cleaves GSDMD and GSDMC, triggering pyroptosis. GSDME: GzmB and caspase-3 cleave GSDME, triggering pyroptosis. GSDMB: GzmA and caspase-1/3/6/7 cleave GSDMB. In addition, the full length GSDMB enhances the caspase-4-mediated GSDMD cleavage to induce pyroptosis. Notes: ASC, apoptosis-associated speck-like protein containing a caspase recruitment domain; PAMP, pathogen-associated molecular pattern; DAMP, damage-associated molecular pattern; IL, interleukin; LPS, lipopolysaccharide; TNF-α, tumor necrosis factor-α; GzmA, granzyme A; GzmB, granzyme B.

Pyroptosis is a form of inflammatory programmed cell death, and is activated by pathogen infections or endogenous danger signals. There are two pathways of pyroptosis: the canonical inflammasome- and caspase-1-dependent pathway, and the noncanonical pathway mediated by caspase-4/5/11 (Figure 1) (Ma et al., 2018). The canonical pathway is initiated by inflammasome-mediated caspase-1 activation. The activated caspase-1 in turn cleaves pro-IL-1β and pro-IL-18 into their active forms. It also cleaves gasdermin D (GSDMD) to generate the GSDMD-N and GSDMD-C domains. GSDMD-N binds to acidic lipids in the cell membrane, forming transmembrane pores that allow IL-1β and IL-18 release, and ion and water influx, resulting in cell swelling and osmotic lysis (You et al., 2023). The noncanonical pathway does not require inflammasome formation. Instead, stimuli such as bacterial lipopolysaccharides (LPS) directly enter the cell to activate caspase-4/5/11, which cleaves GSDMD, and induces pyroptosis. In addition, pyroptosis can be induced by caspase-3-mediated GSDME cleavage, caspase-8-mediated GSDMC cleavage, and caspase-1/3/6/7- or granzyme A-mediated GSDMB cleavage (Liang et al., 2024; Feng et al., 2022; Wang et al., 2017). Although caspase-1 under canonical inflammasome activation has been considered the primary catalytic enzyme for the cleavage of pro-IL-1β and pro-IL-18, recent studies have found that caspase-4, which is a cytosolic receptor for LPS, can also process pro-IL-18 into its active form under non-canonical inflammasome activation (Devant et al., 2023; Shi et al., 2023). This expands the functional significance of caspase-4 in regulating inflammation and immune responses, especially in conditions that involve intracellular pathogens. Pyroptosis is considered a part of the innate immune response that helps eliminate pathogenic microorganisms. Nonetheless, excessive pyroptosis may lead to continuous inflammatory reactions, and cause tissue damage. Pyroptosis has been shown to play a role in the pathogenesis and treatment of a number of human diseases (Jin et al., 2020). In particular, it has emerged as a significant contributing factor to the development of inflammatory respiratory diseases, such as asthma (Feng et al., 2022). This review discusses the current evidence on the involvement of pyroptosis in the development of different inflammatory phenotypes of asthma, the immune and non-immune cells in the airway involved in or affected by pyroptosis, and the potential therapeutic value of targeting pyroptosis for the treatment of specific phenotypes of asthma.

### Immune response to allergens in asthma

Atopic asthma (also known as allergic asthma) is a common type of asthma that is triggered by exposure to allergens, such as dust mites, pollen, or pet dander. It is mostly a type 2 immune disorder characterized by high levels of immunoglobulin E (IgE), and the activation of Th2 cells, mast cells, eosinophils and basophils, as well as non-immune cells, such as airway epithelial cells. The type 2 responses in asthma are regulated by various Th2 cytokines, including IL-4, IL-5, IL-9 and IL-13. Th2 cell-derived IL-4 has been considered to promote IgE production through B cells to specific allergens, which binds to high-affinity FC-ε receptor 1 (FCεR1) on basophils and mast cells. Non-atopic asthma (also known as non-allergic asthma) is less common than atopic asthma. It is triggered by various non-allergic factors, such as air pollution, exercise, infections and stress, and is characterized by the absence of serum IgE, and more involvement of innate immune cells, such as group 2 innate lymphoid cells (ILC2s), basophils and eosinophils. Non-atopic asthma can be triggered by allergenic proteases, which cause a Th2-independent response (Bao et al., 2017; Kubo, 2017; Abu et al., 2020; Jacquet, 2011). Both atopic and non-atopic asthma are characterized by airway inflammation that involve non-immune cells, such as airway epithelial cells (AECs), and immune cells, such as macrophages. Excessive inflammation causes cell death and lung tissue damage that can exacerbate airway inflammation and remodeling. In recent years, accumulating evidence has highlighted the roles of certain types of programmed cell death, such as apoptosis, autophagy and pyroptosis, in the pathogenesis of asthma (Liu et al., 2023a). Pyroptosis, in particular, is an inflammatory form of programmed cell death that can occur in immune and non-immune cells in the inflammatory airway. This article focuses on the involvement of pyroptosis in the pathogenesis and immune response of asthma.

### Immune response to viruses in asthma

People with asthma, especially those with a severe disease, are more susceptible to respiratory viral infections and virus-induced cell cytotoxicity (Sharma et al., 2022; Kim, 2022). Conversely, infections with respiratory viruses, such as influenza, respiratory syncytial virus, and SARS-CoV-2 virus, are a common cause of asthma exacerbation (Yamaya, 2012; Agondi et al., 2022). Factors that lead to increased susceptibility to viral infection in the asthmatic population include impaired airway epithelial barrier function, altered innate immune response, and decreased circulating antibody concentration. AECs are the initial platform of innate immunity against respiratory virus invasion (Kim, 2022). Altered innate immunity of asthmatic AECs is associated with excessive immune response to respiratory infections, causing airway damage and disease aggravation (Kim, 2022). Virus-induced asthma exacerbation may be caused by ineffective virus clearance in asthma patients, leading to persistent infection (Hayashi et al., 2022; Binns et al., 2022). The impaired virus clearance in asthma patients is considered to be caused by altered antiviral immunity that involve eosinophils, Th2 cytokines (e.g., IFN-β and IL-33), and neutrophil extracellular traps (NETs) (Kumar et al., 2020). Given the influence of respiratory viral infection in asthma development and exacerbation, the interaction between viral infection and asthma has become a critical topic in asthma research.

### Pyroptosis in asthma

Increasing evidence has implicated pyroptosis in the development of asthma (Liu et al., 2023a). Single nucleotide polymorphisms (SNPs) in NLRP3 inflammasome and caspase-1 have been linked to the increase in childhood asthma susceptibility (Queiroz et al., 2020). Elevated NLRP3 and caspase-1/4/5 levels have been detected in induced sputum in patients with neutrophilic asthma (Simpson et al., 2014). Mice with ovalbumin (OVA)-induced asthma presented with increased pulmonary NLRP3, caspase-1 and IL-1β expression, while the blockage of the NLRP3/caspase-1/IL-1β pathway attenuated bronchial inflammation in asthmatic mice (Chen et al., 2022). Similarly, NLRP3 inhibition attenuated bronchial inflammation and hyperresponsiveness in asthma mouse models induced by house dust mite (HDM) or toluene diisocyanate (TDI) (Ma et al., 2021; Zhuang et al., 2020). In addition, mice with deficient NLRP3-mediated IL-1β release (NLRP3^−/−^/ASC^−/−^/IL-1R1^−/−^) exhibited reduced bronchial cell infiltration, lung inflammation, and airway hypersensitivity after OVA stimulation (Ritter et al., 2014). Notably, NLRP3-mediated pyroptosis appears to be more prominent in glucocorticoid-resistant, severe, and neutrophilic asthma in humans (Rossios et al., 2018; Kim et al., 2017). These findings indicate that pyroptosis is associated with certain phenotypes of asthma, and the severity of the disease. Thus, pyroptosis inhibitors may provide precision medicine for the personalized treatment of asthma.

### Pyroptosis and non-immune/immune cells in asthma

**FIGURE 2:**
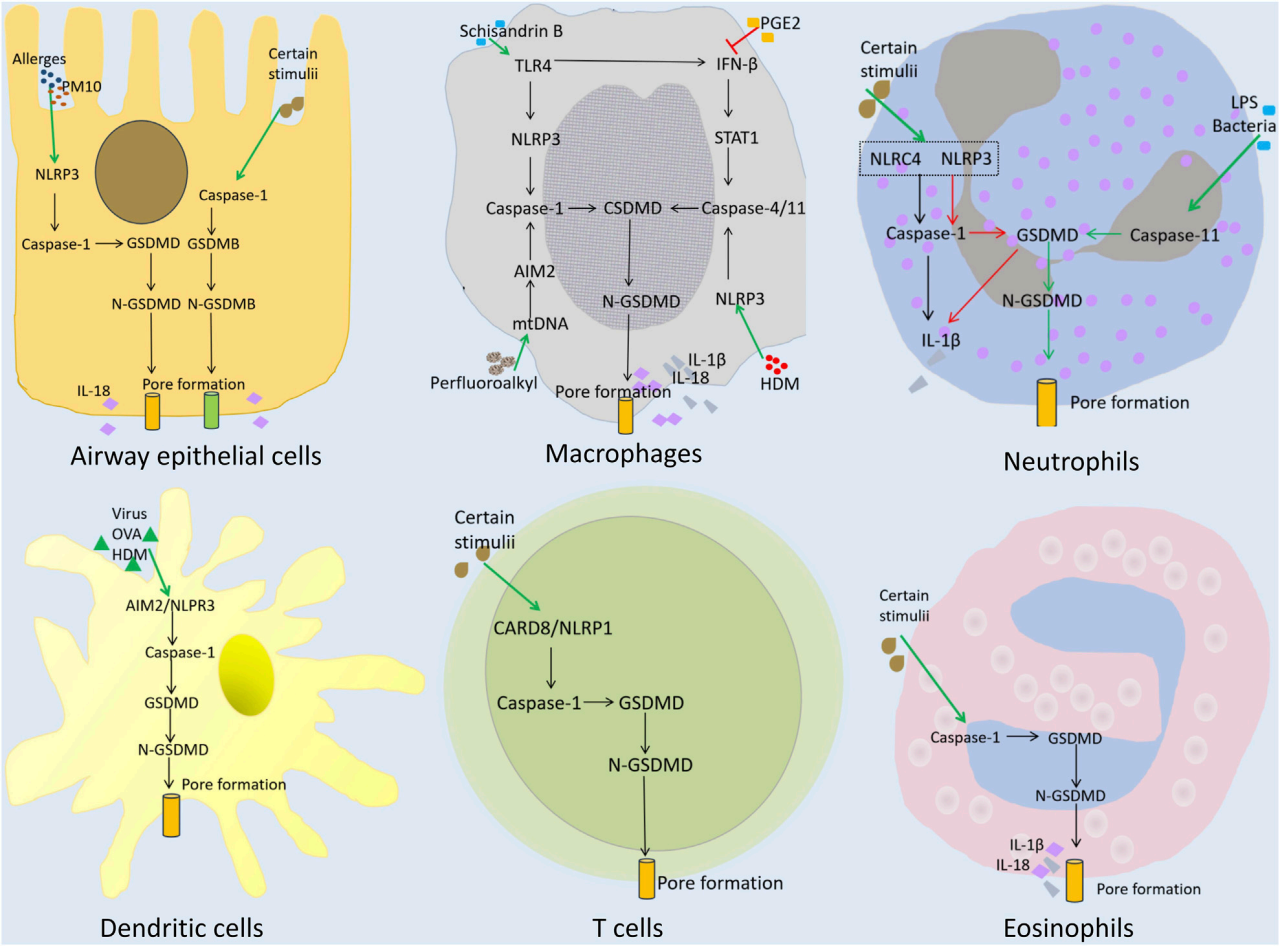
Pyroptosis and the role of immune and non-immune cells in asthma. Airway epithelial cells: Allergens or PM10 activate the NLRP3/caspase-1/GSDMD signaling pathway, triggering pyroptosis. Certain stimuli can activate GSDMB-mediated pyroptosis. Macrophages: Schisandrin B induces TLR4 and IFN-β, leading to the activation of caspase-1 and caspase-4/11, respectively. PGE2 prevents IFN-β induction, and inhibits pyroptosis. Perfluoroalkyl triggers AIM2 inflammasome-mediated pyroptosis through caspase-1. HDM/Curdian triggers pyroptosis through caspase-4/11. Neutrophils: Certain stimuli and LPS/bacteria can trigger caspase-1- or caspase-11/GSDMD-mediated pyroptosis. Dendritic cells and T cells: Caspase-1/GSDMD-mediated pyroptosis. Eosinophils: Caspase-1/GSDMD-mediated pyroptosis.

Pyroptosis in asthma involves and affects various non-immune and immune cells in the airway (Figure 2) (Feng et al., 2022; Sun and Li, 2022; Xiao et al., 2023; Cai et al., 2024). This section reviews how pyroptosis involves/affects different cell types in the airway, and contributes to the development of various inflammatory phenotypes of asthma.

### Pyroptosis of AECs in asthma

The bronchial epithelium primarily functions as a physical barrier, preventing harmful substances, such as pathogens and pollutants, from entering the lungs. It also senses invading substances in the airway, and secrets cytokines and other mediators to boost innate and adaptive immune responses (Cai et al., 2024). AECs express pattern recognition receptors (PRRs), such as toll-like receptors and inflammasomes, which recognize pathogen‐associated molecular patterns (PAMPs) from inhaled microbes/allergens, and damage-associated molecular patterns (DAMPs) from damaged cells. Upon PAMP or DAMP recognition, PRRs trigger the release of various proinflammatory cytokines/chemokines, initiating a cascade of immune responses that help eliminate the invading substances and damaged lung cells. However, the pathogens/allergens themselves, and excessive immune response in the airway can injure AECs, and cause epithelial disruption, leading to impaired barrier function, and increased mucus secretion. This contributes to airway hypersensitivity and inflammation in asthma (Frey et al., 2020). Notably, the AECs of asthma patients are genetically predisposed to an increased barrier permeability (Heijink et al., 2020).

Many studies have implicated AEC pyroptosis in the pathogenesis of asthma. NLRP3 was found to be upregulated in AECs in mice with severe asthma induced by *Chlamydia*/*Haemophilus* respiratory infection combined with OVA challenge (Kim et al., 2017). Particulate matter 10 μm (PM10) can induce NLRP3 inflammasome activation in AECs, leading to cytokine production, dendritic cell activation, and neutrophil recruitment in the lungs (Hirota et al., 2015). Der f1 is an allergen of *Dermatophagoides farina*, which causes pyroptosis in cultured human AECs by activating the NLRP3/caspase-1 pathway (Tsai et al., 2018). In TDI-induced asthmatic mice, TDI induced NLRP3/caspase-1/GSDMD-mediated AEC pyroptosis, and the inhibition of the NLRP3 inflammasome reduced AEC pyroptosis, and attenuated airway inflammation and hyper-responsiveness (Zhuang et al., 2020). In addition, NLRP3-mediated AEC pyroptosis was found to be upregulated in obese asthmatic mice, when compared to control asthmatic mice, contributing to the exacerbation of lung inflammation associated with obesity (Liu F. et al., 2023). Notably, GSDMB is upregulated in the bronchial epithelium of asthma patients, and its overexpression in mice induces airway hyperresponsiveness and remodeling (Das et al., 2016). Furthermore, GSDMB SNPs and expression in AECs correlate to human asthma severity and exacerbations (Li et al., 2021). *In vitro* studies have revealed that GSDMB is cleaved by inflammatory caspase-1 to induce potent AEC pyroptosis (Panganiban et al., 2018). These findings support that GSDMB-mediated bronchial epithelium pyroptosis plays an important role in the pathogenesis of asthma. Thus, the inhibition of NLRP3- or GSDMB-mediated pyroptosis in AECs may reduce asthma severity and exacerbations.

### Pyroptosis of macrophages in asthma

Lung macrophages are the key orchestrators in asthma pathogenesis, in terms of immune cell recruitment, and airway inflammation and remodeling (Britt et al., 2023; Yunna et al., 2020). Exposure to pathogens can induce the polarization of alveolar macrophages into M1 and M2 macrophages. M1 cells express high levels of pro-inflammatory cytokines, such as IL-1β, IL-6 and TNF-α, causing inflammation; and resisting invading pathogens. M2 cells predominantly produce allergic cytokines IL-4, IL-5, IL-9 and IL-13, promoting the migration and infiltration of eosinophils and mast cells, and driving allergic reactions (Jiang and Zhu, 2016). In mouse models of HDM-induced asthma, macrophage pyroptosis mediated by caspase-11/4 stimulated chemokine secretion, and exacerbated airway neutrophil inflammation (Cai et al., 2024; Abu Khweek et al., 2021). In perfluorooctane sulfonate-induced asthmatic mice, macrophage pyroptosis was mediated by the AIM2 inflammasome, rather than the NLRP3 that exacerbates asthmatic lung inflammation through IL-1β (Wang et al., 2021). Prostaglandin E_2_ (PGE_2_) inhibited caspase-11-mediated pyroptosis in murine and human macrophages, and suppressed the experimental allergic bronchial inflammation triggered by OVA or HDM (Abu Khweek et al., 2021; Zaslona et al., 2020). Notably, blocking NLRP3/caspase-1/GSDMD-mediated macrophage pyroptosis with Schisandrin B attenuated bronchial inflammation and remodeling in OVA-induced asthmatic rats (Chen et al., 2021). These findings support macrophage pyroptosis as a key contributing factor to the development of asthma, and that blocking this process may provide therapeutic benefits to asthma patients.

### Pyroptosis and neutrophils in asthma

Neutrophilic inflammation is a pathological hallmark of neutrophilic and mixed granulocytic asthma. Elevated NLRP3 and caspase 1/4/5 levels were detected in the induced sputum of patients with neutrophilic asthma, when compared to patients with eosinophilic asthma (Simpson et al., 2014). The inhibition of the NLRP3 inflammasome can prevent the severe bronchial neutrophilic inflammation triggered by fungal allergen *Alternaria alternata* in mice (Killian et al., 2023). These findings indicate that inflammasome activation is involved in neutrophilic inflammation in asthma. Neutrophil extracellular traps (NETs) are a network of extracellular DNA strips and proteins released from activated or dying neutrophils. These are present in the airway of asthma patients, and help to eliminate invading pathogens. However, excessive NET formation (NETosis) can cause airway damage, accelerating asthma progression (Poto et al., 2022). Indeed, high NETs in induced sputum is a marker of severe asthma and inflammasome activation (Lachowicz-Scroggins et al., 2019). Studies have revealed that caspase 11-driven and GSDMD-dependent neutrophil pyroptosis is responsible for NETosis, in response to LPS stimulation or bacterial infection (Chen et al., 2018). Thus, blocking the GSDMD cleavage can prevent the neutrophil pyroptosis and NETosis triggered by phorbol 12-myristate 13-acetate (PMA) (Sollberger et al., 2018). Notably, the activation of the NLRP3/caspase-1/GSDMD signaling pathway in neutrophils, in response to adenosine triphosphate (ATP) or microbial toxin pneumolysin, can lead to IL-1β secretion, without inducing pyroptosis (Mankan et al., 2012; Karmakar et al., 2016; Karmakar et al., 2020; Karmakar et al., 2015). Similarly, the activation of NLRC4/caspase-1 signaling in neutrophils after *Salmonella* infection can drive IL-1β production, but not pyroptosis (Chen et al., 2014). This resistance to pyroptotic cell death keeps neutrophils alive to continuously produce IL-1β, and sustain neutrophilic inflammation, which may function as a mechanism that drives the progression of severe, glucocorticoid-resistant neutrophilic asthma (Kim et al., 2017).

### Pyroptosis and dendritic cells (DCs) in asthma

As dominant antigen-presenting cells, DCs play a central role in initiating and sustaining Th2 immune responses in the airways of asthma patients (Morianos and Semitekolou, 2020). There have been few studies on the pyroptosis of DCs in the development of asthma. DCs can undergo pyroptosis in antiviral defense or allergic inflammation. For example, immune-complexed adenovirus can engage the DNA PRR AIM2 to induce caspase-1/GSDMD-dependent pyroptosis in human DCs (Eichholz et al., 2016). Furthermore, OVA/HDM stimulation can induce NLRP3/GSDMD-mediated pyroptosis in DCs *in vitro*, affecting CD4^+^ T cell differentiation, and causing Th1/Th2/Th17 cell imbalance in a co-culture system (Qiao et al., 2023). In mice with OVA-induced allergic rhinitis, it was found that DCs, but not mast cells or basophils, underwent GSDMD-mediated pyroptosis. Blocking the GSDMD-mediated pyroptosis can prevent Th1/Th2/Th17 imbalance, and alleviate OVA-induced inflammatory responses, while the adoptive transfer of OVA-stimulated DCs would exacerbate OVA-induced inflammation (Qiao et al., 2023). Intriguingly, inflammasome activation in conventional type 2 DCs (DC2) does not activate pyroptosis, but instead induces the secretion of IL-12 family cytokines and IL-1β, which in turn activates potent Th1 and Th17 responses (Hatscher et al., 2021). In contrast, inflammasome activation in type 3 DCs (DC3) causes pyroptotic cell death. Thus, the ability of DC2 to circumvent pyroptosis upon inflammasome activation renders DC2 greater potency, when compared to DC3, in inducing Th1/Th17 response (Hatscher et al., 2023). These *in vitro* findings suggest that DC subsets may contribute in different ways to the development of asthma due to its varying susceptibility to pyroptosis.

### Pyroptosis and eosinophils in asthma

Eosinophils play an essential role in the key clinical manifestations of asthma, from airway hyperresponsiveness and inflammation, to mucus hypersecretion (McBrien and Menzies-Gow, 2017). Caspase-1- or NLRP3-deficient mice exposed to HDM exhibited enhanced eosinophil recruitment, and exacerbated airway inflammation (Madouri et al., 2015), suggesting that the NLPR3/caspase-1 axis restrain eosinophilic inflammation in asthma. However, the blockade of the NLRP3/caspase-1 axis attenuated the bronchial neutrophilic inflammation in mice exposed to toluene diisocyanate (TDI) (Chen et al., 2019), implying that the NLPR3/caspase-1 signaling promotes neutrophilic inflammation in asthma. Thus, the role of NLRP3/caspase-1 signaling in asthma appears to depend on the inflammatory phenotype of asthma. There have been few studies on the pyroptosis of eosinophils in asthma. Necrotic liver injury can induce massive eosinophilia, accompanied by caspase-1-mediated eosinophil pyroptosis, degranulation, and IL-1β/IL-18 secretion (Palacios-Macapagal et al., 2017). IL-1β and IL-18 contribute to the development of allergic inflammatory diseases by activating eosinophil recruitment, and stimulating mast cell degranulation (Sanders and Mishra, 2016; Wan et al., 2015; Ge et al., 2021; Wang Y. et al., 2024). It is possible that in the development of asthma, the IL-1β and IL-18 secreted from eosinophils that undergo pyroptosis help drive the progression of airway hypersensitivity and inflammation. Further investigations are needed to confirm this hypothesis.

### Pyroptosis and T cells in asthma

CD4^+^ T cells are the main determinant of the inflammatory phenotype of asthma. Th2, Th9 and Tfh cells drive the development of eosinophilic asthma, while Th1 and Th17 cells control the progression of neutrophilic asthma (Jeong and Lee, 2021). There have been few studies on T cell pyroptosis in the development of asthma. The massive IL-18 and IL-1β release from pyroptotic AECs and macrophages may influence the differentiation, proliferation and function of CD4^+^ T cells, and determine the course of asthma progression (Huber et al., 1996; Ben-Sasson et al., 2009; Smith, 2011). IL-18 can induce FasL expression in natural killer and cytotoxic T cells, promoting Fas-mediated epithelial apoptosis and tissue damage during inflammatory responses (Dinarello, 1998). Furthermore, IL-18 can promote IL-4-independent IgE production mediated by CD4^+^ T cells (Hoshino et al., 2000). In mice with allergic asthma, it was found that intrapulmonary IL-18 administration can increase Th2 cytokine levels, IgE production, eosinophil influx, and airway mucus secretion (Wild et al., 2000). Small molecule inhibitors of dipeptidyl peptidases 8 and 9 can activate CARD8 and NLRP1 inflammasomes in T cells, causing caspase‐1‐GSDMD-mediated pyroptosis (Johnson et al., 2020; Linder et al., 2020; Linder and Hornung, 2022). However, it remains to be determined whether T cells undergo pyroptosis during the development of asthma.

### Pyroptosis and intercellular communication in asthmatic inflammation

**FIGURE 3:**
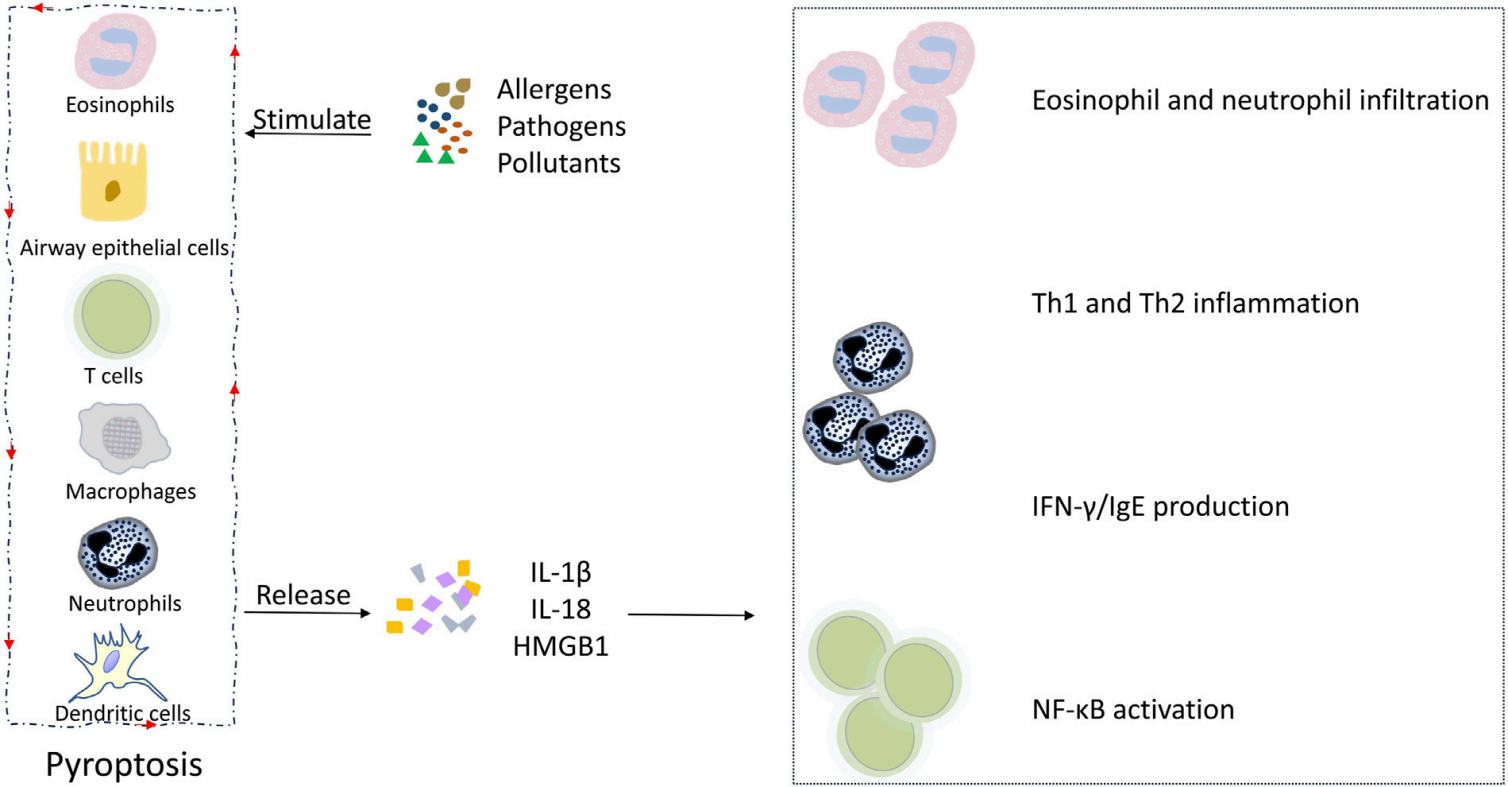
Pyroptosis and its intercellular communication in asthmatic inflammation. Allergens, pathogens, and pollutants induce the pyroptosis of AECs and airway macrophages, leading to the release of IL-1β and IL-18, which in turn promotes eosinophil and neutrophil infiltration, Th1 and Th2 inflammation, INF-γ and IgE production, and NF-κB activation, contributing to the clinical manifestations of asthma. Meanwhile, immune cell-derived inflammatory factors, such as TNF-α, enhance the pyroptosis of AECs, creating a positive feedback loop that amplifies the inflammatory cascade.

Asthmatic inflammation involves both the sequential and intricate interactions of non-immune and immune cells that contribute to the initiation and propagation of an inflammatory cascade. Pyroptosis is a component of this complex cascade, which leads to clinical manifestations associated with the disease. Inhaled allergens, pathogens, and pollutants can directly activate the pyroptosis of AECs and immune cells in the airway, resulting in the release of IL-1β, IL-18, and high mobility group box 1 (HMGB1). IL-1β plays an important role in asthmatic inflammation, contributing to eosinophilia, IgE switching, and Th2 inflammation (Linder and Hornung, 2022). IL-18 can induce the infiltration of neutrophils and eosinophils in the lungs, causing airway hyperresponsiveness (Sawada et al., 2013). IL-18 can also exacerbate airway inflammation by promoting Th1 and Th2 cell activation, and IFN-γ and IgE production (Sawada et al., 2013; Lu et al., 2024; Xu et al., 2023). HMGB1 can promote NF-κB activation and the consequent recruitment of effector T cells, causing the aggregation of CD8^+^ T cells in the airway epithelium (Frey et al., 2020). Meanwhile, immune cell-derived inflammatory factors, such as TNF-α, can enhance the pyroptosis of AECs, creating a positive feedback loop that amplifies the inflammatory cascade. Figure 3 illustrates the crosstalk between non-immune and immune cells bridged by pyroptosis in asthmatic inflammation. At present, the research in this field remains rather limited, and there are various gaps in current knowledge, especially in how immune cell-derived factors affect non-immune cell pyroptosis. Future studies are needed to fill these gaps.

### Pyroptosis and asthma treatment

Inhaled corticosteroids are the preferred medication for treating asthma. However, some patients, especially patients with neutrophilic asthma, may develop glucocorticoid resistance. In recent years, a number of biologics and small molecules that target pyroptosis pathways have presented with therapeutic benefits in *in vitro* and *in vivo* models of asthma (Table 1). JT002, which is a small molecule inhibitor of the NLRP3 inflammasome, inhibited pyroptosis, and alleviated airway hyperresponsiveness and neutrophilia in a mouse model of IL-17-dependent neutrophilic asthma (Ambrus-Aikelin et al., 2023). Schisandrin B, which is a bioactive biphenycloctadine isolated from *Schisandra chinensis*, exhibits diverse antioxidant and anti-inflammatory properties (Nasser et al., 2020). Furthermore, Schisandrin B reduced bronchial inflammation and remodeling in asthmatic rats (Chen et al., 2021). Mechanistically, Schisandrin B inhibited NLRP3 activation, and reduced pyroptosis in asthmatic rats and LPS-stimulated rat alveolar macrophages via the miR-135a-5p/TRPC1/STAT3/NF-κB axis (Chen et al., 2021). The C‐terminal subunit of mucin 1 (MUC1‐CT) was found to inhibit NLRP3-dependent pyroptosis by downregulating the TLR4/MyD88/NF-κB signaling, thereby reducing bronchial neutrophilic inflammation in OVA/LPS-stimulated asthmatic mice (Liu et al., 2023c). Nootkatone, which is an antioxidant sesquiterpenoid identified in a number of *Citrus* species, alleviated bronchial inflammation and mucus hypersecretion in mice with OVA-induced asthma (Gai et al., 2023) Mechanistically, Nootkatone mitigated the ROS-mediated NLRP3 activation and pyroptosis in asthmatic mice, and in IL-13-stimulated human AECs (Gai et al., 2023). Substance P activated the PI3K/Akt/NF-κB signaling to promote inflammation and NLRP3/caspase-1-dependent pyroptosis in cultured human AECs and asthmatic mice *in vitro* and *in vivo*, and aggravated bronchial asthma in OVA-induced mice (Li et al., 2022). Blocking substance P receptor neurokinin-1 receptor (NK1R), PI3K, or NF-κB can protect against substance P-induced AEC inflammation and pyroptosis (Linder et al., 2020). Yanghe Pingchuan granules (YPG) are a traditional Chinese herbal medicine preparation used to treat bronchial asthma in China (Li et al., 2022). YPG inhibits airway smooth muscle cell pyroptosis, and alleviates bronchial asthma by downregulating the TLR4/NF-κB/NRLP3 signaling (Pan et al., 2022). Protopine, which is an anti-inflammatory isoquinoline alkaloid obtained from plants, ameliorates OVA-induced asthma by downregulating the TLR4/MyD88/NF-κB signaling, and reducing NLRP3-mediated pyroptosis (Yang et al., 2024). These results support that medications that target inflammasomes and pyroptosis may provide novel treatments for asthma, especially severe, glucocorticoid-resistant asthma.

**TABLE 1:** Biologics and small molecules that mitigate asthma by targeting pyroptosis.

Biologics and small molecules	Targets	Animal models of asthma	Efficacy	References
JT002	NLRP3	Mouse model of neutrophilic asthma	Reduced airway hyperresponsiveness and airway neutrophilia	Ambrus-Aikelin et al. (2023)
Schisandrin B	NLRP3	OVA/acetylcholine chloride-induced asthmatic rats	Alleviated airway inflammation and remodeling	Chen et al. (2021)
MUC1-CT	TLR4/MyD88/NF-κB/NLRP3	OVA/LPS-stimulated asthmatic mice	Reduced neutrophilic airway inflammation	Liu et al. (2023c)
Nootkatone	NLRP3	OVA-induced asthmatic mice	Mitigated oxidative stress and airway inflammation	Gai et al. (2023)
L732138	NK1R	OVA/Substance P-induced asthmatic mice	Suppressed pro-inflammatory cytokine secretion	Li et al. (2022)
YPG	TLR4/NF-κB/NRLP3	OVA-induced asthmatic rats	Alleviated lung inflammation	Pan et al. (2022)
Protopine	TLR4/MyD88/NF-κB/NLRP3	OVA-induced asthmatic rats	Ameliorated inflammation and pathological changes in the lungs	Yang et al. (2024)

Emerging evidence has implicated the E3 ligase TRIM29 in the regulation of virus-induced pyroptosis of intestinal epithelial cells (IECs). TRIM29 suppresses rotavirus and encephalomyocarditis virus-induced pyroptosis of human IECs by targeting the NLRP6 and NLRP9b inflammasomes for ubiquitination-mediated degradation (Wang et al., 2024b). Furthermore, TRIM29 can promote the virus-induced pyroptosis of porcine IECs through protein kinase RNA-like endoplasmic reticulum kinase (PERK)-mediated endoplasmic reticulum (ER) stress (Ma et al., 2024; Wang et al., 2024c). Moreover, it was found that the RNA helicase DEAH-box helicase 15 (DHX15) activates the NLRP6 inflammasome and IL-18 production during viral infection in mouse IECs (Xing et al., 2021). Given the important roles of AECs and respiratory viral infection in asthma development and exacerbation, TRIM29 and DHX15 modulators may mitigate asthma by influencing AEC pyroptosis and immune response during viral infection.

## Conclusion

Accumulating evidence has indicated that pyroptosis contribute to the pathogenesis of asthma. During the development of asthma, both non-immune cells, such as AECs, and immune cells, such as macrophages, neutrophils, eosinophils and T cells, may undergo pyroptosis through the inflammasome-mediated, caspase-1-dependent canonical pathway or caspase-4/5/11-dependent noncanonical pathway, resulting in the secretion of proinflammatory cytokines and DAMP molecules. This helps to initiate and sustain inflammatory responses, such as immune cell infiltration, T cell differentiation and activation, and airway mucus hypersecretion, influencing the progression of asthma. Present evidence supports that medications that target inflammasomes and pyroptosis may provide novel treatments for asthma, especially severe, glucocorticoid-resistant asthma.

## Abbreviations

ATP, adenosine triphosphate; DCs, dendritic cells; DC2, type 2 DCs; DC3, type 3 DCs; DAMP, damage-associated molecular pattern; FCεR1, FC-εreceptor 1; GSDMD, gasdermin D; HDM, house dust mite; HMGB1, high mobility group protein 1; IgE, immunoglobulin E; ILC2s, innate lymphoid cells; NETs, neutrophil extracellular traps; NETosis, NET formation; OVA, ovalbumin; PRRs, pattern recognition receptors; PAMPs, pathogen-associated molecular patterns; PGE2, Prostaglandin E2; PMA, phorbol 12-myristate 13-acetate; SNP, single nucleotide polymorphism; TDI, toluene diisocyanate.
